# Hyphal Growth of Phagocytosed *Fusarium oxysporum* Causes Cell Lysis and Death of Murine Macrophages

**DOI:** 10.1371/journal.pone.0101999

**Published:** 2014-07-15

**Authors:** Katja Schäfer, Judith M. Bain, Antonio Di Pietro, Neil A. R. Gow, Lars P. Erwig

**Affiliations:** 1 Departamento de Genética, Universidad de Córdoba, Córdoba, Spain; 2 Aberdeen Fungal Group, Institute of Medical Sciences, University of Aberdeen, Aberdeen, United Kingdom; King's College London Dental Institute, United Kingdom

## Abstract

*Fusarium oxysporum* is an important plant pathogen and an opportunistic pathogen of humans. Here we investigated phagocytosis of *F. oxysporum* by J774.1 murine cell line macrophages using live cell video microscopy. Macrophages avidly migrated towards *F. oxysporum* germlings and were rapidly engulfed after cell-cell contact was established. *F. oxysporum* germlings continued hyphal growth after engulfment by macrophages, leading to associated macrophage lysis and escape. Macrophage killing depended on the multiplicity of infection. After engulfment, *F. oxysporum* inhibited macrophages from completing mitosis, resulting in large daughter cells fused together by means of a *F. oxysporum* hypha. These results shed new light on the initial stages of *Fusarium* infection and the innate immune response of the mammalian host.

## Introduction

*Fusarium* species cause devastating diseases on a wide variety of economically important crops worldwide [1]. In addition, *Fusaria* can cause a broad spectrum of diseases in humans, ranging from superficial or localized infections in immunocompetent hosts to lethal disseminated fusariosis in immunocompromised patients [2].

Previous work established that the tomato pathogenic isolate *F. oxysporum* f. sp. *lycopersici* can cause disseminated infection in immunosupressed mice, making this the first fungal model for studying fungal pathogenicity across different host kingdoms [3], [4]. *F. oxysporum* has been studied in detail as a plant pathogen and is attracting increasing interest as a model for cross-kingdom pathogenicity in fungi [5]. Analysis of knockout mutants in the mouse model revealed striking similarities of infection mechanisms with other well-established human pathogens [3], [5], [6], [7], [8].

The early events of the infection process and host defence mechanisms in the *Fusarium*-mouse interaction are currently unknown. The mammalian immune response against human fungal pathogens relies mainly on phagocytosis of the fungus by cells of the innate immune system [9], [10]. Phagocytic clearance of fungal pathogens can be classified into distinct stages (reviewed in [11]): recognition of pathogen-associated molecular pattern (PAMPs) and migration towards fungal cells; cell-cell contact and engulfment of fungal cells bound to the phagocyte cell membrane; phagosome maturation and processing of engulfed cells within the phagocyte; and killing of the phagocyte by the fungus. In *C. albicans*, phagocyte killing is associated with hyphal growth within the macrophage [12], [13]. The invasive properties of fungal hyphae promote the escape from immune cells resulting in death of the phagocytes [12], [14], [15], whereas the yeast form promotes dissemination in the bloodstream [16]. Murine macrophage phagocytosis displays strong preferences based on genus, species and morphology. For example, *C. albicans* yeast cells are engulfed preferentially compared to hyphal cells [13], [17].

In this study we investigated phagocytosis of the filamentous fungus *F. oxysporum* by the J774.1 murine macrophage cell line. Hyphae of this pathogen have been shown to penetrate mammalian tissues and to cause invasive fusariosis in different organs of immunosupressed mice [4]. Here we used live cell video microscopy coupled with image analysis tools to obtain detailed insights into the major stages of the phagocytosis process, including migration, engulfment and phagocyte killing. This detailed step-by-step analysis has been reported previously only for *C. albicans* [13]. We found that germinated microconidia of *F. oxysporum* undergo rapid uptake by murine macrophages, once cell-cell contact is established. Fungal hyphae continue growth within the phagosome, ultimately leading to their escape from the macrophages and to host cell lysis. This pattern of events resembles that of other more common human pathogens, but also has some unique features.

## Materials and Methods

### *F. oxysporum* isolate and culture conditions

*F. oxysporum* f.sp. *lycopersici* wild-type strain 4287 (FGSC 9935) was originally obtained from J. Tello, University of Almeria, Spain and stored as a glycerol microconidial suspension at -80°C. *Fusarium* conidia were cultured in potato dextrose broth at 28°C and 150 rpm for 4 days. For preparation of macrophage assay, microconidia were isolated by filtration as described previously [18], collected by centrifugation, washed, and resuspended in DMEM medium (Lonza, Slough, UK). Conidia suspensions were counted using a haemocytometer and adjusted to a final concentration of 1.5×10^6^/ml.

### *F. oxysporum* preparation and staining with fluorescein isothiocyanate (FITC)

To visualize *F. oxysporum* during phagocytosis assays, germlings were harvested and stained with 1 mg/ml freshly prepared cell membrane label FITC (Sigma, Dorset, UK) dissolved in 0.05 M carbonate-bicarbonate buffer (pH 9.6) (BDH Chemicals, VWR International, Leicestershire, United Kingdom) for 20 min at room temperature in the dark. After centrifuging at 3,000×g for 5 min the supernatant was removed and the pellet was washed three times in 1 ml of 1× PBS to remove unbound FITC before the pellet was finally resuspended in 1× DMEM (Lonza, Slough, UK).

### Culturing and preparation of the J774.1 mouse macrophage cell line

J774.1 macrophage cell cultures (ECACC, HPA, Salisbury, UK) were maintained in 75 cm^2^ tissue culture flasks (Nagle Nunc. International, Hereford, UK) in Dulbecco's Modified Eagle medium (DMEM; Lonza, Slough, UK) supplemented with 10% (v/v) fetal calf serum (FCS; Biosera, Ringmer, UK), 200 U/ml penicillin/streptomycin (Invitrogen, Ltd., Paisley, UK) and 2 mM L-glutamine (Invitrogen) at 37°C with 5% CO_2_. Macrophages were scraped from the tissue culture flask and transferred to a 50 ml Falcon tube and centrifuged at 600×g for 5 min to obtain a cell pellet. Supernatant was removed and the pellet was resuspended in 10 ml pre-warmed supplemented DMEM medium (Lonza, Slough, UK). Cells were counted using a haemocytometer.

### J774.1 macrophage cell preparation for live cell imaging

A total of 1×10^6^ J774.1 macrophages were plated in 2 ml supplemented DMEM medium in a 35 mm glass-based Iwaki imaging dish (VWR, Leicestershire, UK) and incubated overnight at 37°C, 5% CO_2_. Prior to imaging, supplemented DMEM medium was replaced with 2 ml pre-warmed supplemented CO_2_-independent medium (Gibco, Invitrogen, Paisley, UK; with 10% (v/v) fetal calf serum (FCS), 200 U/ml penicillin/streptomycin and 2 mM L-glutamine) containing the phagosome staining dye 1 µM LysoTracker Red DND-99 (Invitrogen, Paisley, UK).

### Live Cell Video microscopy phagocytosis assay

Phagocytosis assay with *F. oxysporum* was performed using a protocol previously described for *C. albicans* [13], [19]. *F. oxysporum* microconidia (6×10^5^ c/ml) were germinated for 8 h in DMEM medium at 37°C with 5% CO_2_, stained with FITC (as described above) and added at a 3∶1 ratio to a glass-based Iwaki imaging dish containing macrophages stained with LysoTracker Red (described above) in supplemented CO_2_-independent medium (Gibco, Invitrogen, Paisley, UK). Video microscopy was performed at 37°C with a DeltaVision Core microscope (Applied Precision, Washington, USA) and images captured at 1 min intervals for 6 h by an EMCCD camera.

### Analysis of live cell video microscopy movies

Murine macrophages were imaged and recorded by video-microscopy and uptake events were analyzed individually at 1 min intervals throughout the 6 h phagocytosis assay. Macrophage migratory responses to the presence of *F. oxysporum* were determined by tracking directional and distance components of movement between 1 min intervals for the first 30 min of live video microscopy movies, as this represents a period of elevated migratory activity [13]. Volocity 6.3.0. software (PerkinElmer, Massachusetts, USA) was used to track and analyze 50 macrophages from 3 representative movies. The rate of engulfment of *F. oxysporum* cells by macrophages (n = 219) were determined by the time points at which an *F. oxysporum* cell was fully engulfed, defined as the time taken from establishment of cell-cell contact to complete ingestion of an *F. oxysporum* cell [13]. A fungal cell was considered to have been fully ingested when the FITC fluorescent signal was diminished, indicating that the fungal cell was inside the macrophage [20]. Measurements taken included *F. oxysporum* uptake, defined as the number of *F. oxysporum* germlings taken up by an individual phagocyte (n = 190) over a 6 h period. The percentage of macrophage killing was defined as the percentage of macrophages (n = 194) that had been killed by specific time points over a 6 h period. Counting was used to calculate the percentage of macrophages killed by *F. oxysporum* in relation to the defined number of phagocytosed germlings over a 6 h period.

### Ethics statement

All animal experimentation was done in accordance with UK Home Office regulations and was approved by both the UK Home Office and the University of Aberdeen ethical review committee.

## Results

### *F. oxysporum* maintains hyphal growth after engulfment, resulting in lysis of phagocytes and fungal escape

**Figure 1:**
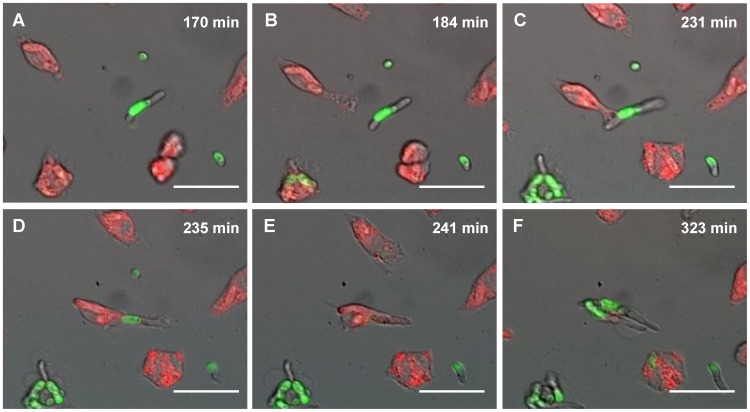
Engulfment of a *F. oxysporum* germling by a J774.1 macrophage, followed by fungal outgrowth and macrophage lysis. Snapshots taken from live cell video microscopy capturing the engulfment process of a *F. oxysporum* germling by a macrophage (A–F). A macrophage (red) and a *F. oxysporum* germling (green) are shown prior to cell-cell contact (A); at time of phagocyte recognition (B); during cell-cell contact (C); during phagocytosis (D); after engulfment (E); and after outgrowth of *F. oxysporum* leading to macrophage cell lysis (F). Scale bar, 20 µm.

To investigate the interaction between *F. oxysporum* and J774.1 macrophages, we established a macrophage phagocytosis assay using live cell video microscopy, as previously described for *C. albicans* [13], [19]. We found that macrophages of this cell line efficiently take up *F. oxysporum* germlings (Video S1). Here we examined different stages of the phagocytosis assay consisting of migration, engulfment and fungal escape, followed by macrophage cell lysis (presented in snapshots Fig. 1A–F, see also Video S1).

**Figure 2:**
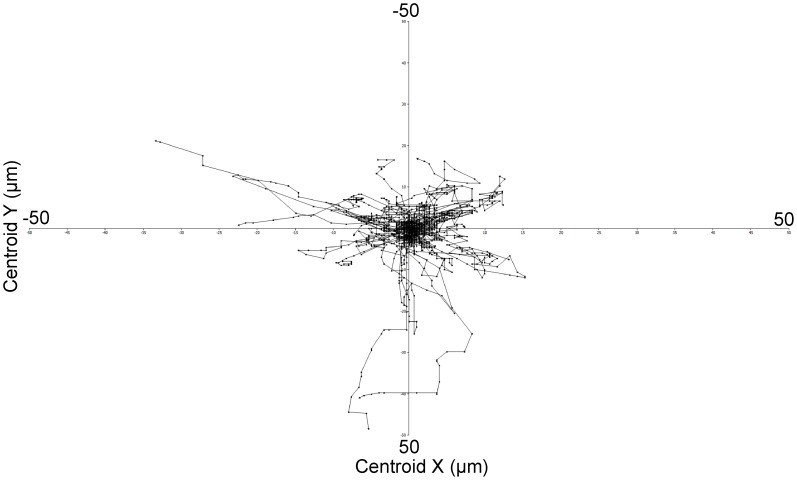
Macrophage migration towards *F. oxysporum* germlings. Tracking diagram shows a detailed dissection of macrophage migration dynamics illustrating the distances travelled, directionality and velocity of J774.1 macrophages cultured with *F. oxysporum* germlings. Tracks represent the movement of individual macrophages (n = 50) relative to their starting position and symbols indicate the location at 1 min intervals.

Efficient uptake of fungal cells requires migration of phagocytes towards the target [13]. The migration kinetics of 50 macrophages was determined and the tracks plotted relative to their starting position (Fig. 2) to indicate directionality and distance traveled assessed in 1 min intervals. Track data were used to measure mean track velocity which was 1.22 µm min^−1^ above random baseline migration of macrophages (macrophages not subsequently engulfing fungi). Previous studies defined the baseline velocity of the same macrophage cell line as 1.8 µm min^−1^ in the absence of fungal particles [13], thus the mean track velocity of macrophages in response to *F. oxysporum* is 3.1 µm min^−1^ (n = 50).

**Figure 3:**
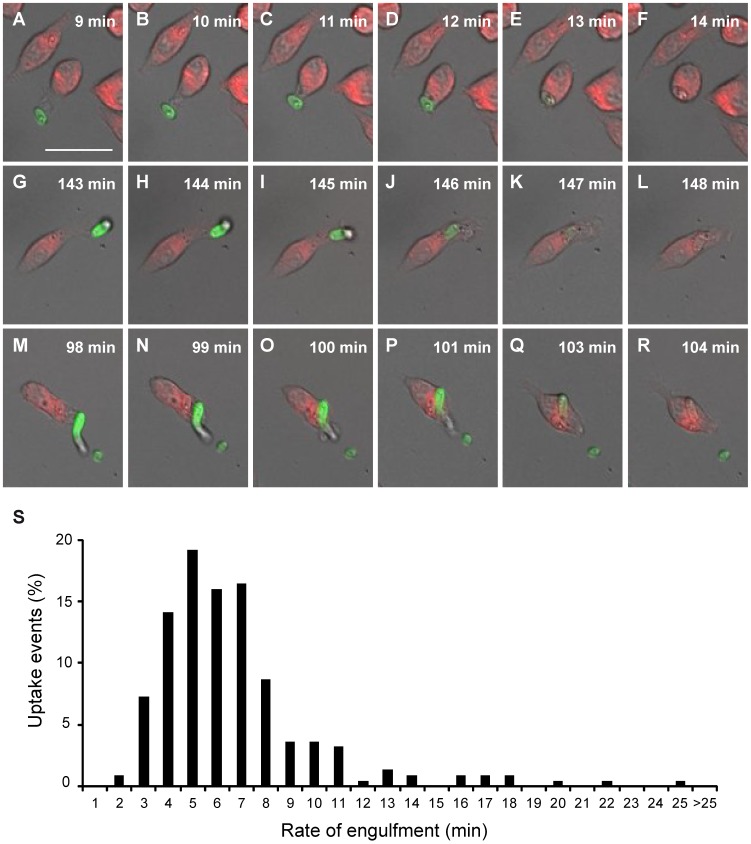
Time of macrophage engulfment of live *F. oxysporum* germlings. Snapshots of live cell video microscopy showing 3 events (A–F; G–L; M–R) of various stages of *F. oxysporum* phagocytosis by J774.1 macrophages during phagocytosis assay. (A, G, M) Macrophages (red) and *F. oxysporum* germlings (green) establishing cell-cell contact, (B–E; H–K and N–Q) initializing of fungal cell engulfment; (F; L and R) *F. oxysporum* inside the macrophages after engulfment. The times in the Figures A–F; G–L and M–R showing the time (min) taken for J774.1 macrophages to ingest *F. oxysporum* cells following cell-cell contact. (S) Times taken for phagocytosis of *F. oxysporum* germlings by macrophages. The rate of engulfment was defined as the time taken from first cell-cell contact to complete ingestion of *F. oxysporum* cells by the phagocytes. Bars represent the percentage of uptake events. Scale bar, 20 µm.

Macrophage migration towards fungal particles is necessary to establish fungal cell contact, the rate of engulfment, defined as the time elapsed between the establishment of cell-cell contact and the complete uptake of the fungus [13]. We used live cell video microscopy and subsequent image analysis to generate a detailed minute-by-minute account of the engulfment process. Fig. 3A–R shows exemplary events of migration of a macrophage towards a *F. oxysporum* germling (germinated microconidia) and its subsequent engulfment. *F. oxysporum* germlings were rapidly phagocytosed by macrophages, once cell-cell contact was established, the average engulfment time being 6.74 min (n = 219) (Fig. 3S). The vast majority (93%) of *F. oxysporum* cells that became bound to a macrophage were taken up within the first 11 min. None of the germlings were internalized within less than 2 min or more than 25 min (Fig. 3S).

**Figure 4:**
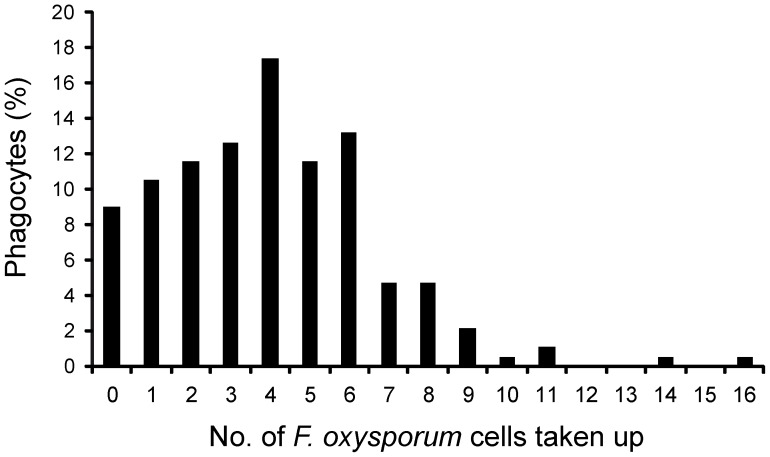
Numbers of *F. oxysporum* cells ingested by J774.1 macrophages. Bars represent percentage of macrophages that engulfed a defined number of *F. oxysporum* cells at the end of the phagocytosis assay. The *F. oxysporum* cell/phagocyte ratio was 3∶1. The majority of *F. oxysporum* germlings were engulfed rapidly by macrophages once cell-cell contact was established.

The total number of *F. oxysporum* cells taken up by individual macrophages was recorded over a 6 h period. An uptake event was defined as the complete engulfment of one *F. oxysporum* germling by one macrophage cell following cell-cell contact. Most of the macrophages (80.5%, n = 190) ingested more than one fungal cell (Fig. 4). For example, video S2 shows the internalization of 14 *F. oxysporum* germlings by the central macrophage within the first 160 minutes. Following engulfment, growth of hyphal filaments within the macrophage was observed. The membrane of the macrophage frequently failed to restrain hyphal expansion, resulting in rupture and lysis of the phagocyte. At this point, the fluorescence of the FITC labeled fungal germlings became visible again (visible at 196 min in Video S2). Macrophage cell lysis was accompanied by rapid appearance of a bubble-like structure (visible at 230 min in Video S2), followed by extensive hyphal growth of *F. oxysporum* and disappearance of the macrophage. After escaping from a macrophage fungal hyphae were recognized by other macrophages, which initiated engulfment until they were lysed themselves by the fungal hyphae (Video S2).

### The rate of macrophage killing increases with the number of internalized *F. oxysporum* cells

**Figure 5:**
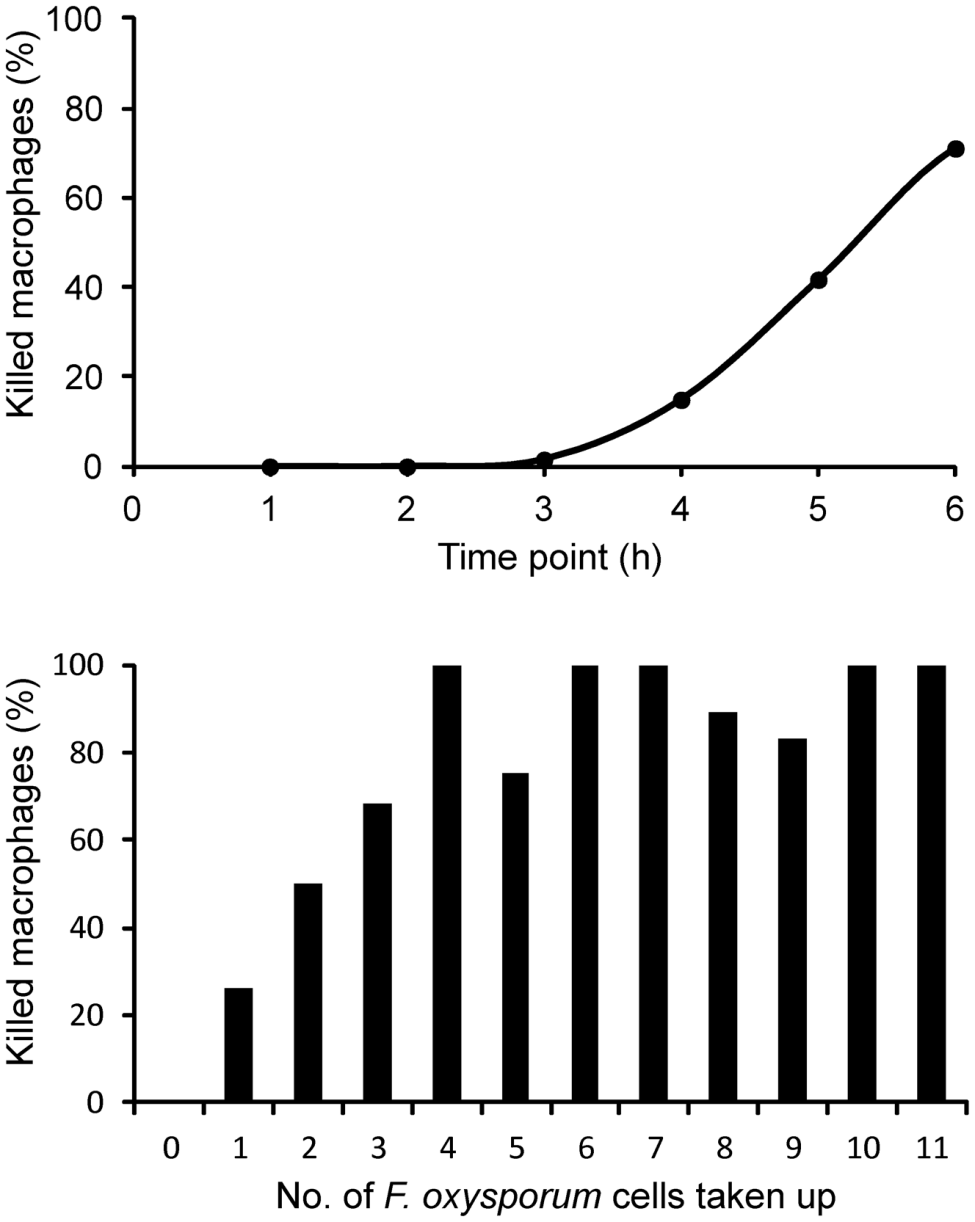
*F. oxysporum* mediated killing of J774.1 murine macrophages. (A) Percentage of macrophages killed by *F. oxysporum* germlings over a 6 h period. The viability was defined as the percentage of macrophages that had been killed by specific time points. (B) Percentage of macrophages killed by *F. oxysporum* in relation to the defined number of phagocytosed germlings over a 6 h period.

*F. oxysporum* initialized phagocyte lysis 3 h after engulfment (Fig. 5A). Lysis increased over time, causing death of 71% of the macrophages after 6 h. We observed a linear increase in phagocyte killing in relation with the number of internalized fungal cells (Fig. 5B). A very high fraction (93.4%) of the macrophages that took up 4 or more fungal germlings was killed within the 6 h of observation. In contrast, killing was less than 50% for macrophages which took up 3 or less fungal cells (Fig. 5B). Interestingly, a small fraction (13.8%) of the macrophages survived 6 h even after ingesting up to 9 fungal germlings, although almost all of them died before 8 h. Thus, the number of engulfed fungal cells plays a crucial role in killing of J774.1 macrophages.

### Macrophages with phagocytosed *F. oxysporum* germlings inhibit mitosis

**Figure 6:**
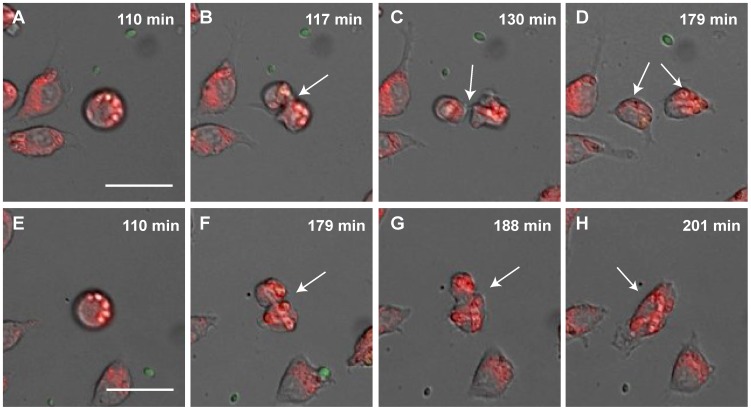
Successful and failed mitosis of J774.1 macrophage cells after engulfment of *F. oxysporum*. The macrophages (A and E) initiated mitosis with phagocytosed *F. oxysporum* germlings (B and F). This resulted either in successfully completed mitosis with the appearance of two separated daughter cells (C–D and Video S3), or failure of the macrophages to complete cell separation (G–H). In the latter case, the macrophages initiated mitosis (F) but instead of completely separating, the daughter cells remained fused together (G) by means of a *F. oxysporum* hypha spanning both cells (F and G). The two daughter cells then fuse to reform a single cell (H and Video S4). Scale bar, 20 µm.

We used live cell video microscopy to follow the dynamics of macrophage mitosis. During the observation period, almost 10% of the J774.1 macrophage cells with phagocytosed *F. oxysporum* germlings initiated mitosis (n = 384). This is lower as in previous studies which showed that 30.8% of the same macrophage cell line underwent mitosis in the absence of fungal cells [21]. Mitosis was successfully completed in 74% of the cases (n = 28), as determined by the appearance of two separate daughter cells (Fig. 6A–D, Video S3). After completing mitosis, the daughter cells continued engulfment of fungal cells until they were lysed by the phagocytosed germlings (Video S3). In the remaining 26% of the macrophages that had initiated mitosis, the two daughter cells remained together by means of a *F. oxysporum* hypha spanning both cells, and subsequently fused back into a large single cell (Fig. 6E–H, Video S4).

## Discussion

A key aspect of virulence in filamentous fungal pathogens is the capacity for penetration and dissemination, which is a prerequisite for systemic infection. Macrophages are key components of the innate immune response in mammals and provide an important line of defense against fungal invaders by directly engulfing and destroying fungal cells (reviewed in [11]).

Both, *F. oxysporum* and the airborne fungus *A. fumigatus* are opportunistic human pathogens characterized by filamentous hyphal growth. *A. fumigatus* conidia are inhaled and exposed to attack by alveolar macrophages (AMs), the major phagocytes present in lung alveoli along with polymorphonuclear neutrophils (PMNs) [22], [23], [24], [25]. *Aspergillus* conidia that escape from AM can then germinate, but are attacked by PMNs which kill the hyphae through production of reactive oxygen species and degranulation [26], [27], [28].

In contrast to *Aspergillus*, *Fusarium* enters the human body mostly through the skin, to reach the bloodstream [29]. Here we analyzed the interaction between *F. oxysporum* germinated microconidia (germlings) and J774.1 macrophages. To our knowledge, this is the first analysis of the phagocytosis process in the important opportunistic pathogen *Fusarium*. Our results demonstrate that murine macrophages efficiently migrate towards and internalize *F. oxysporum* germlings. The use of video microscopy allowed a detailed dissection of these processes, revealing remarkable similarities with the results previously reported for *C. albicans* [13]. However the migration velocity of macrophages at 3.1 µm min^−1^ is faster compared to those values obtained previously for J774.1 macrophages responding to *C. albicans* strains, ranging from 2.2–2.7 µm min^−1^ [13]. The average engulfment time of *F. oxysporum* (6.74 min) was almost identical to that reported in *C. albicans* (6.7 min). Likewise, the fraction of fungal cells bound to a macrophage that were taken up after 15 min was also very similar (96% and 95% for *F. oxysporum* and *C. albicans*, respectively [13]. We found that the number of engulfed fungal germlings crucially affected the survival of the macrophage. A very large fraction (93.4%) of the macrophages that internalized 4 or more germlings were killed by *F. oxysporum* whereas less than 50% of those were killed that engulfed less than 4 germlings. Since most of the macrophages (61.8%) ingested more than 3 germlings, a large fraction of these eventually succumbed to the fungus. In our experiments it appeared that the vast majority of J774.1 macrophage cell death was driven by hyphal mediated piercing of the macrophage cell membrane rather than by the recently described mechanism of pyroptosis [30].

*F. oxysporum* hyphae that had escaped from the killed macrophage were subsequently engaged and engulfed, often by multiple macrophages (Video S2). In spite of multiple macrophages simultaneously trying to engulf, phagocytosis of large hyphae was frequently frustrated (see exemplary Video S2), suggesting a limitation for successful phagocytosis with increasing hyphal length, similar to what has been reported for *C. albicans* [21].

Mitosis of tissue-derived macrophages plays an important role in macrophage proliferation. Inhibition of macrophage cell division was previously reported for the fungal pathogens *Cryptococcus neoformans*, *Candida krusei* and *C. albicans* [21], [31], [32]. Here we found that in the presence of *F. oxysporum*, mitosis of macrophages was unsuccessful in approximately 25% of the cases. This proportion is similar, although somewhat lower than that reported in *C. albicans* (35,9%) [21]. Strikingly, the percentage of macrophages underwent mitosis in presence of *F. oxysporum* (9.9%) was lower than previously reported for the same macrophage cell line in the absence of fungal particles (30.8%) or cultured with *C. albicans* (29.5%) [21]. It has been suggested that interference of the fungus with macrophage cell division may inhibit the formation of new uninfected macrophages. On the other hand, however, successful mitosis of macrophages carrying fungal cells may also contribute to the spreading of the pathogen within the host [21].

Although phagocytosis of *F. oxysporum* may be crucial to protect the host, the mechanisms and molecules involved in this process remain unknown. Recognition and phagocytosis of *C. albicans* by macrophages is dependent on the glycosylation status and specific components of the fungal cell wall [12]. Likewise, conidial germination in *A. fumigatus* is associated with an increase of β1,3-glucan in the outer cell wall. Because β1,3-glucans are targeted by the pattern-recognition receptor Dectin-1 which is expressed in macrophages, monocytes, neutrophils and a subset of T cells [33], [34], germ tubes are recognized more efficiently than ungerminated conidia [35] leading to phagocytosis and synthesis of different proinflammatory cytokines [36]. In contrast to germinated conidia, *A. fumigatus* resting conidia are immunologically inert. These dormant conidia are covered by a surface “rodled layer” a thin coating of regularly arranged RodA hydrophobins [37] which cap immune-triggering structures of the fungal surface preventing both innate and adaptive immune response [38]. It has been suggested that the lack of recognition of rodlet protein by the immune system is a universal phenomenon from airborne conidia of filamentous fungi [38].

Currently, little is known about the cell wall components of *F. oxysporum* modulating recognition and uptake by macrophages, as well as the role of these surface molecules in the ability of the fungus to evade destruction by immune cells. Our results highlight the need for more detailed studies on the interaction between *F. oxysporum* and the mammalian immune system, which will lead to a better understanding of the early molecular events during *Fusarium* infection.

## Supporting Information

Video S1 **Overview of the phagocytosis assay of** ***F. oxysporum*** **and J774.1 murine macrophages.** Shown is a representative 6 h live video microscopy of *F. oxysporum* germlings being engulfed by macrophages and *F. oxysporum* hyphal growth within macrophages leading to killing through lysis.
(AVI)

Video S2 **Phagocytosis of** ***F. oxysporum*** **by J774.1 murine macrophages.** Shown is a live video of a representative phagocytosis event. The central macrophage ingests up to 14 *F. oxysporum* germlings. Hyphal growth within the macrophages can be observed leading to macrophage cell lysis. The escaped fungal hyphae are recognized by multiple macrophages which then begin hyphal engulfment.
(AVI)

Video S3 **Successful mitosis of a J774.1 macrophage after engulfment of** ***F. oxysporum*** **.** Shown is a live video of a macrophage which successfully undergoes mitosis. The central macrophage containing phagocytosed *F. oxysporum* germlings initiates and successfully completes mitosis, shown by the appearance of two separated daughter cells.
(AVI)

Video S4 **Failed mitosis of a J774.1 macrophage after engulfment of** ***F. oxysporum*** **.** Live video of a macrophage which fails to complete cell separation. The macrophage containing phagocytosed *F. oxysporum* germlings initiates mitosis (130 min). Instead of completely separating, the two daughter cells remain attached together by means of a *F. oxysporum* hypha spanning both cells, and eventually the two daughter cells fuse back into a single cell.
(AVI)
